# Hereditary cerebellar degeneration and stem-cell based therapy

**DOI:** 10.1186/1878-5085-5-S1-A141

**Published:** 2014-02-11

**Authors:** Pavel Ostasov, Milena Kralickova, Jan Cendelin, Jan Tuma, Yaroslav Kolinko

**Affiliations:** 1 Department of Histology and Embryology, Faculty of Medicine in Pilsen, Charles University in Prague, Pilsen, Czech Republic; 2 Biomedical Centre, Faculty of Medicine in Plzen, Charles University in Prague, Plzen, Czech Republic; 3 Department of Pathophysiology, Faculty of Medicine in Pilsen, Charles University in Prague, Pilsen, Czech Republic

## 

Hereditary cerebellar ataxias are a wide group of progressive, degenerative diseases, which manifest as lack of control and coordination of voluntary movements due to progressive loss of Purkinje cells and other cell types in the cerebellum. Cerebellar degeneration could affect people of any age, is often diagnosed after symptoms start to manifest and no prevention and no causal and really effective therapy is available today.

There is high variability in pathogenesis in human cerebellar degenerations and high variability in onset and progress of the disease. Though the basal manifestation of all cerebellar degenerations is similar as it is related to dysfunction of the cerebellum, there are some differences between individual types of them and also some variability in the frame of one disease making the clinical diagnosis difficult. The manifestation of the mutation could be also modified by the genetic background. For all of that, individual approach of treatment is necessary.

There is a vide spectrum of mouse models of genetically determined cerebellar degenerations including both spontaneous mutants and transgenic mice, some of which are available in several mouse strains. This situation is analogous to variability of human hereditary cerebellar disorders and genetic heterogeneity of human population. Mouse models can be used for investigation of efficiency of experimental therapy in individual types of cerebellar degenerations and for studying of the effect of genetic background on the pathological process. Such research could allow identifying factors determining progress of the disease and influencing the effect of the therapy. This is necessary to choose the optimal treatment for each individual patient and for development of new therapeutic methods.

One of therapies which could benefit from such research is stem cell and neurotransplantation therapy. So far it seems promising in slowing down progress, but it might even mitigate neurological deficit caused by the cerebellar degeneration.

Nevertheless, as experiments in mice shoved different types of grafts have different effects. For example mesenchymal stem cells prevent neurodegeneration progression, but do not reverse already developed cell loss and neurological deficit. Transplanted suspension of embryonic cerebellar cells generates new graft-derived Purkinje cells in Purkinje cell degeneration mice and improvement of motor function was described in them. On the other hand, in Lurcher mice functional benefit of cerebellar tissue transplantation has not been achieved yet. In addition, cultivated embryonic neural stem cells survive well in the host cerebellum and despite they do not create any new Purkinje cells they have been shown to rescue Purkinje cells in a mouse model of Niemann-Pick disease.

Genetically defined mouse strains with different mutations causing neurodegenerative diseases and different mouse strain backgrounds for the same mutations and analysis of their genome and proteome would be valuable tool to identify genetic markers and factors important for assessment of prognosis and management of appropriate stem-cell therapy and for development of new therapeutic approaches based on the principles of personalized medicine.

